# Quality assurance of qualitative research: a review of the discourse

**DOI:** 10.1186/1478-4505-9-43

**Published:** 2011-12-19

**Authors:** Joanna Reynolds, James Kizito, Nkoli Ezumah, Peter Mangesho, Elizabeth Allen, Clare Chandler

**Affiliations:** 1 Department of Global Health & Development, London School of Hygiene & Tropical Medicine, London, UK; 2 Infectious Diseases Research Collaboration, Mulago Hospital Complex, Kampala, Uganda; 3 Department of Sociology/Anthropology, University of Nigeria, Nsukka, Nigeria; 4 National Institute for Medical Research, Amani Centre, Muheza, Tanzania; 5 Division of Clinical Pharmacology, Department of Medicine, University of Cape Town, Cape Town, South Africa

**Keywords:** Qualitative, global health, quality, quality assurance, guidance, meta-narrative, literature review

## Abstract

**Background:**

Increasing demand for qualitative research within global health has emerged alongside increasing demand for demonstration of quality of research, in line with the evidence-based model of medicine. In quantitative health sciences research, in particular clinical trials, there exist clear and widely-recognised guidelines for conducting quality assurance of research. However, no comparable guidelines exist for qualitative research and although there are long-standing debates on what constitutes 'quality' in qualitative research, the concept of 'quality assurance' has not been explored widely. In acknowledgement of this gap, we sought to review discourses around quality assurance of qualitative research, as a first step towards developing guidance.

**Methods:**

A range of databases, journals and grey literature sources were searched, and papers were included if they explicitly addressed quality assurance within a qualitative paradigm. A meta-narrative approach was used to review and synthesise the literature.

**Results:**

Among the 37 papers included in the review, two dominant narratives were interpreted from the literature, reflecting contrasting approaches to quality assurance. The first focuses on demonstrating quality within research outputs; the second focuses on principles for quality practice throughout the research process. The second narrative appears to offer an approach to quality assurance that befits the values of qualitative research, emphasising the need to consider quality throughout the research process.

**Conclusions:**

The paper identifies the strengths of the approaches represented in each narrative and recommend these are brought together in the development of a flexible framework to help qualitative researchers to define, apply and demonstrate principles of quality in their research.

## Background

The global health movement is increasingly calling for qualitative research to accompany its projects and programmes [1]. This demand, and the funding that goes with it, has led to critical debates among qualitative researchers, particularly over their role as applied or theoretical researchers [2]. An additional challenge emanating from this demand is to justify research findings and methodological rigour in terms that are meaningful and useful to global public health practitioners. A key area that has grown in quantitative health research has been in quality assurance activities, following the social movement towards evidence-based medicine and global public health [3]. Through the eyes of this movement, the quality of research affects not only the trajectory of academic disciplines but also local and global health policies. Clinical trials researchers and managers have led much of health research into an era of structured standardised procedures that demarcate and assure quality [4,5].

By contrast, disciplines using qualitative research methods have, to date, engaged far less frequently with quality assurance as a concept or set of procedures, and no standardised guidance for assuring quality exists. The lack of a unified approach to assuring quality can prove unhelpful for the qualitative researcher [6,7], particularly when working in the global health arena, where research needs both to withstand external scrutiny and provide confidence in interpretation of results by internal collaborators Furthermore, past and existing debates on what constitutes 'good' qualitative research have tended to be centred firmly within social science disciplines such as sociology or anthropology, and as such, their language and content may prove difficult to penetrate for the qualitative researcher operating within a multi-disciplinary, and largely positivist, global health environment.

The authors and colleagues within the ACT Consortium [8] conduct qualitative research that is mostly rooted in anthropology and sociology, to explore the use of antimalarial medicines and intervention trials around antimalarial drug use, within the global health field. Through this work, within the context of clinical trials following Good Clinical Practice (GCP) guidelines [4], we have identified a number of challenges relating to the demands for evidence of quality and for quality assurance of qualitative research. The quality assurance procedures available for quantitative research, such as GCP training and auditing, are rooted in a positivist epistemology and are not easily translated to the reflexive, subjective nature of qualitative research and the interpretivist-constructionist epistemological position held by many social scientists, including the authors. Experiences of spatial distance between collaborators and those working in remote study field sites have also raised questions around how best to ensure that a qualitative research study is being conducted to high quality standards when the day-to-day research activity is unobservable by collaborators.

In response to the perceived need for the authors' qualitative studies to maintain and demonstrate quality in research processes and outcomes, we sought to identify existing guidance for quality assurance of qualitative research. In the absence of an established unified approach encapsulated in guidance format, we saw the need to review literature addressing the concept and practice of quality assurance of qualitative research, as a precursor to developing suitable guidance.

In this paper, we examine how quality assurance has been conceptualised and defined within qualitative paradigms. The specific objectives of the review were to, firstly, identify literature that expressly addresses the concept of quality assurance of qualitative research, and secondly, to identify common narratives across the existing discourses of quality assurance.

## Methods

### Search strategy

Keywords were identified from a preliminary review of methodological papers and textbooks on qualitative research, reflecting the concepts of 'quality assurance' and 'qualitative research', and all their relevant synonyms. The pool of keywords was augmented and refined iteratively as the search progressed and as the nature of the body of literature became apparent. Five electronic databases-Academic Search Complete, CINAHL Plus, IBSS, Medline and Web of Science-were searched systematically between October and December 2010, using combinations of the following keywords: "quality assurance", "quality assess*", "quality control*", "quality monitor*", "quality manage*, "audit*", "quality", "valid*", "rigo*r", "trustworth*", "legitima*", "authentic*", "strength", "power", "reliabil*", "accura*","thorough*", "credibil*", "fidelity", "authorit*", "integrity", "value", "worth*", "good*", "excellen*", "qualitative AND (research OR inquiry OR approach* OR method* OR paradigm OR epistemolog* OR study). Grey literature was also searched for using Google, and the key phrases "quality assurance" AND "qualitative research".

Several relevant journals-*International Journal of Qualitative Methods, International Journal of Social Research Methodology *and *Social Science and Medicine *- were hand searched for applicable papers using the same keywords. Finally, additional literature, in particular books and book chapters, was identified through snowballing techniques, both backwards by following references of eligible papers and forwards through citation chasing. At the point where no new references were identified from the above techniques, the decision was made to curtail the search and begin reviewing, reflecting the practical and time implications of adopting further search strategies.

### Inclusion and exclusion criteria

Inclusion criteria were identified prior to the search, to include:

Methodological discussion papers, books or book chapters addressing qualitative research with explicit focus on issues of assuring quality.

Guidance or training documents (in 'grey literature') addressing quality assurance in qualitative research.

Excluded were:

Publications primarily addressing critical appraisal or evaluation of qualitative research for decision-making, reviews or publication. These topics were considered to be distinct from the activity of quality assurance which occurs before writing up and publication.

Publications focusing only on one or more specific qualitative methods or methodological approaches, for example grounded theory or focus groups; focusing on a single stage of the research process only, for example, data collection; or primarily addressing mixed methods of qualitative and quantitative research. It was agreed by the authors that these method-specific papers would not help inform narratives about the discourse of quality assurance, but may become useful at a later date when developing detailed guidance.

Publications not in the English language.

### Review methodology

A meta-narrative approach was chosen for the reviewing and synthesis of the literature. This is a systematic method developed by Greenhalgh et al [9] to make sense of complex, conflicting and diverse sources of literature, interpreting the over-arching narratives across different research traditions and paradigms [9,10]. Within the meta-narrative approach, literature is mapped in terms of its paradigmatic and philosophical underpinnings, critically appraised and then synthesised by constructing narrative accounts of the contributions made by each perspective to the different dimensions of the topic [9]. Due to the discursive nature of the literature sought, representing different debates and philosophical traditions, the meta-narrative approach was deemed most appropriate for review and synthesis. A process of evaluating papers according to predefined quality criteria and using methods to minimise bias, as in traditional, Cochrane-style systematic reviewing, was not considered suitable or feasible to achieve the objectives.

Each paper was read twice by JR, summarised and analysed to determine the paper's academic tradition, the debates around quality assurance in qualitative research identified and discussed, the definition(s) used for 'quality' and the values underpinning this, and recommended methods or strategies for assuring quality in qualitative research. At the outset of the review, the authors attempted to identify the epistemological position of each paper and to use as a category by which to interpret conceptualisations of quality assurance. However, it emerged that fewer than half of the publications explicitly presented their epistemology; consequently, epistemological position was not used in the analytical approach to this review, but rather as contextual information for a paper, where present.

Following the appraisal of each paper individually, the literature was then grouped by academic disciplines, by epistemological position (where evident) and by recommendations. This grouping enabled the authors to identify narratives across the literature, and to interpret these in association with the research question. The narratives were developed thematically, following the same process used when conducting thematic analysis of qualitative data. First, the authors identified key idea units in each of the papers, then considered and grouped these ideas into broader cross-cutting themes and constructs. These themes, together with consideration of the epistemologies of the papers, were then used to develop overarching narratives emerging from the reviewed literature.

## Results

### Search results

The above search strategy yielded 93 papers, of which 37 fulfilled the inclusion and exclusion criteria on reading the abstracts or introductory passages. Of the 56 papers rejected, 26 were papers specifically focused on the critical evaluation or appraisal of qualitative research for decision-making, reviews or publication. The majority of the others were rejected for focusing solely on guidance for a specific qualitative method or single stage of the research process, such as data analysis. Dates of publication ranged from 1994 to 2010. This relatively short and recent timeframe can perhaps be attributed in part to the recent history of publishing qualitative research within the health sciences. It was not until the mid-1990s that leading medical publications such as the *British Medical Journal *began including qualitative studies [11,12], reflecting an increasing acknowledgement of the value of qualitative research within the predominant evidence-based medicine model [13,14]. Within evidence-based medicine, the emphasis on assessment of quality of research is strong, and as such, may account for the timeframe in which consideration of assuring quality of qualitative research emerged.

Among the 37 papers accepted for inclusion in the review, a majority, 19, were from the fields of health, medical or nursing research [6,15-32]. 11 papers represented social science in broad terms, but most commonly from a largely sociological perspective [33-43]. Three papers came from education [44-46], two from communication studies [47,48] and one each from family planning [49] and social policy [50]. In terms of the types of literature sourced, there were 27 methodological discussion papers, 3 papers containing methodological discussion with one case study, two editorials, two methodology books, two guidance documents and one paper reporting primary research.

### Appraisal of literature

#### Epistemological positions

In only 10 publications were the authors' epistemological positions clearly identifiable, either explicitly stated or implied in their argument. Of these publications, five represented a postpositivist-realist position [16,24,39,44,47], and five represented an interpretive-constructionist position [17,21,25,34,38]; see Table 1 for further explanation of the authors' use of these terms. Many of the remaining publications appeared to reflect a postpositivist position due to the way in which authors distinguished qualitative research from positivist, quantitative research, and due to the frequent use of terminology derived from Lincoln and Guba's influential postpositivist criteria for quality [51].

**Table 1:** Defining epistemology and qualitative research

Epistemology:	
***Definition***	***Relating to quality***

Epistemology reflects the relationship between the inquirer and that which is to be known [60]. An epistemological approach entails assumptions about what is to be considered knowledge and the appropriate ways to construct or produce it.	Epistemology is crucial to defining what one considers constitutes quality in research: research questions, methods and interpretations all depend upon epistemological assumptions.

**Qualitative epistemological approaches identified in debates around quality:**	

***Postpositivism/realism***	***Interpretivism/constructionism***

Social reality exists but can never be fully apprehended, only approximated [18]. Methods must be systematic and rigorous [45].	Reality does not exist; knowledge is constructed through the research process and interpreted through the researcher's own values and assumptions [27].

### Narratives

Two strong narratives across the body of literature were interpreted through the review process, plus one other minor narrative.

#### Narrative 1: quality as assessment of output

A majority of the publications reviewed (n = 22) demonstrated, explicitly or implicitly, an evaluative perspective of quality assurance, linked to assessment of quality by the presence of certain indicators in the research output [15,16,18-22,24,26,27,30,32,36,39,40,42,44,45,47-50]. These publications were characterized by a 'post-hoc' approach whereby quality assurance was framed in terms of demonstrating that particular standards or criteria have been met in the research process. The publications in this narrative typically offered or referred to sets of criteria for research quality, listing specific methods or techniques deemed to be indicators of quality, and the documenting of which in the research output would be assurance of quality [15,18-20,24,26,32,39,42,47,48,50].

##### Theoretical perspectives of quality

Many of the authors addressing quality of qualitative research from the output perspective drew upon recent debates that juxtapose qualitative and quantitative research in efforts to increase its credibility as an epistemology. Several of the earlier publications from the 1990s discussed the context of an apparent lack of confidence in quality of qualitative research, particularly against the rising prominence of the evidence-based model within health and medical disciplines [16,19,27]. This contextual background links into the debate raised in a number of the publications around whether qualitative research should be judged by the same constructs and criteria of quality as quantitative research.

Many publications engaged directly with the discourse of the post-positivist movement of the mid-1980s and early 1990s to develop criteria of quality unique to qualitative research, recognizing that criteria rooted in the positivist tradition were inappropriate for qualitative work [18,20,24,26,39,44,47,49,50]. The post-positivist criteria developed by Lincoln and Guba [51], based around the construct of 'trustworthiness', were referenced frequently and appeared to be the basis upon which a number of authors made their recommendations for improving quality of qualitative research [18,26,39,47,50]. A number of publications explicitly drew on a post-positivist epistemology in their approach to quality of qualitative research, emphasising the need to ensure research presents a 'valid' and 'credible' account of the social reality [16,18,24,39,44,47]. In addition, a multitude of other, often rather abstract, constructs denoting quality were identified across the literature contributing to this narrative, including: 'rigour', 'validity', 'credibility', 'reliability', 'accuracy', 'relevance', 'transferability' 'representativeness', 'dependability' and more.

##### Methods of quality assurance

Checklists of quality criteria, or markers of 'best practice', were common within this output-focused narrative [15,16,19,20,24,32,39,42,47,48], with arguments for their value centring on a perceived need for standardised methods by which to determine quality in qualitative research [20,42,50]. Typically, these checklists comprised specific techniques and methods, the presence of which in qualitative research, was deemed to be an indicator of quality. Among the publications that did not proffer checklists by which to determine quality, methodological techniques signalling quality were also prominent among the authors' recommendations [26,40,44,49].

A wide range of techniques were referenced across the literature in this narrative as indicators of quality, but common to most publications were recommendations for the use of triangulation, member (or participant) validation of findings, peer review of findings, deviant or negative case analysis and multiple coders of data. Often these techniques were presented in the publications with little explanation of their theoretical underpinnings or in what circumstances they would be appropriate. Furthermore, there was little discussion within the narrative of the quality of these techniques themselves, and how to ensure they are conducted well.

##### Recognition of limitations

Two of the more recent papers in this review highlight debates of a more fundamental challenge around defining quality, linked to the challenges in defining the qualitative approach itself [26,32]. These papers, and others, reflect upon the plethora of different terminology and methods used in discourse around quality in qualitative research, as well as the numerous different checklists and criteria available to evaluate quality [20,32,40,42]. Some critique is offered of the inflexibility of fixed lists of criteria by which to determine quality, with authors emphasizing that standards, and the corresponding techniques by which to achieve them, should be selected in accordance with the epistemological position underpinning each research study [18,20,22,30,32,45]. However, in much of the literature there is little guidance around how to determine which constructs of quality are most applicable, and how to select the appropriate techniques for its demonstration.

#### Narrative 2: assuring quality of process

The second narrative identified was less prominent than the first, with fewer publications addressing the assurance of quality in terms of the research process (n = 13). Among these, several explicitly stated the need to consider how to assure quality through the research process, rather than merely evaluating it at output stage [6,17,31,33,34,37,38,43]. The other papers addressed aspects of good qualitative research or researcher that could be considered process rather than output-oriented, without explicitly defining them as quality assurance methods [23,25,35,41,46]. These included process-based methods such as recommending the use of field diaries for on-going self-reflection [25], and researcher-centred attributes such as an 'underlying methodological awareness' [46].

##### Theoretical perspectives of quality

Conceptualisations of quality within the literature contributing to this narrative appeared most commonly to reflect a fundamental, internal set of values or principles indicative of the qualitative approach, rather than theoretical constructs such as 'validity' more traditionally linked to the positivist paradigm. These were often presented as principles to be understood and upheld by the research teams throughout the research process, from designing a study, through data collection to analysis and interpretation [17,31,34,37,38]. Six common principles were identified across the narrative: reflexivity of the researcher's position, assumptions and practice; transparency of decisions made and assumptions held; comprehensiveness of approach to the research question; responsibility towards decision-making acknowledged by the researcher; upholding good ethical practice throughout the research; and a systematic approach to designing, conducting and analyzing a study.

Of the four papers in this narrative which explicitly presented an epistemological position, all represented an interpretive/constructionist approach to qualitative research. These principles reflected the prevailing argument in this narrative that unthinking application of techniques or rules of method does not guarantee quality, but rather an understanding of and engagement with the values unique to qualitative paradigms are crucial for conducting quality research [6,25,31].

##### Critique of output-based approach

Within this process-focused narrative emerged a strong theme of critique of the approach to evaluating quality of qualitative research by the research output [6,17,25,31,33,35,37,38,43,46]. The principle argument underpinning this theme was that judging quality of research by its output does not help assure or manage quality in the process that leads up to it, but rather, the discussion of what constitutes quality should be maintained throughout the research [43,46]. Furthermore, several papers explicitly criticised the use of set criteria or standards against which to determine the quality of qualitative research [6,34,37,46], arguing that checklists are inappropriate as they may fail to accommodate the subjectivity and creativity of qualitative inquiry. As such, many studies may appear lacking or of poor quality against such criteria [46].

A number of authors within this narrative argued that checklists can promote the 'uncritical' use of techniques considered indicative of quality research, such as triangulation. Meeting specific criteria may not be a true indication of the quality of the activities or decisions made in the research process [37,43] and methodological techniques become relied upon as *"technical fixes" *[6] which do not automatically lead to good research practice or findings. Authors argued that the promotion of such checklists of may result in diminished researcher responsibility for their role in assuring quality throughout the research process [6,25,35,38], leading to a lack of methodological awareness, responsiveness and accountability [38].

##### Assuring quality of the research process

A number of activities were identified across this narrative to be used along the course of qualitative research to improve or assure its quality. They included the researcher conducting an audit or decision trail to document all decisions and interpretations made at each stage of the research [25,33,37]; on-going dynamic discussion of quality issues among the research team [46]; and developing reflexive field diaries in which researchers can explore and capture their own assumptions and biases [17]. Beyond these specific suggestions, however, were only broader, more conceptual recommendations without detailed guidance on exactly how they could be enacted. These included encouraging researchers to embrace their responsibility for decision making [38], understanding and applying a broad understanding of the rationale and assumptions behind qualitative research [6], and ensuring that the 'attitude' with which research is conducted, as well as the methods, are appropriate [37].

Although specific recommendations to assure quality were not present in all papers contributing to this narrative, there were some commonalities across each publication in the form of the principles or values that the authors identified as underpinning good quality qualitative research. Some of the publications made explicit reference to principles of good practice that should be appreciated and followed to help assure good quality qualitative research, including transparency, comprehensiveness, reflexivity, ethical practice and being systematic [6,25,35,37]. Across the other publications in this narrative, these principles emerged from definitions or constructs of quality [34], from recommendations of strategies to improve the research process [17,31,38,43], or through critiques of the output-focused approach to evaluating quality [33].

#### Minor narrative

Two papers did not contribute coherently to either of the two major narratives, but were similar in their approach towards addressing quality of qualitative research [28,29]. Both were methodological discussion papers which engaged with recent and ongoing debates around quality of qualitative research. The authors drew upon the plurality of views of quality within qualitative research, and linked it to the qualitative struggle to demonstrate credibility alongside quantitative research [29], and the contested nature of qualitative research itself [28].

The publications also shared critique of existing discourse around quality of qualitative research, but without presentation of alternative ways to assure it. Both papers critiqued the output-focused approach, conceptualising quality in terms of the demonstration of particular technical methods. However, neither paper offers a clear interpretation of the process of quality assurance; when and how it should be conducted, and what it should seek to achieve. One paper synthesised other literature and described abstract principles of qualitative research that indicate quality, but it was not clear whether these were principles were intended as guidance for the research process or standards against which to evaluate the output. Similarly, the second paper argues that quality cannot be assured by predetermined techniques, but does not offer more constructive guidance. Perhaps it can be said that these two papers encapsulate the difficulties that have been faced within the qualitative research field with defining quality and articulating appropriate ways to assure that it reflects the principles of the qualitative approach, which itself is contested.

#### Synthesis of the two major narratives

The key features of the two major narratives emerging from the review, assuring quality by output and assuring quality by process, have been captured in Table 2. This table details the perspectives held by each approach, the context in which the narratives are situated, how quality is conceptualised, and examples from the literature of recommended ways in which to assure quality.

**Table 2:** Two dominant narratives identified in the literature on assuring quality of qualitative research

Narrative	Perspective	Context	Conceptualisation of quality in qualitative research	Examples	Methods for quality assurance recommended in the literature
**Output-oriented approach**	External, post-hoc	Efforts to demonstrate credibility of research alongside dominant positivist paradigm, often in context of evidence-based medicine model	Range of theoretical constructs of quality; drawn from positivist paradigm, or post-positivist theory	Validity Rigour Confirmability Credibility Trustworthiness	Demonstrating use of techniques considered to be indicators of quality practice, for example: • triangulation • member checking • negative case analysis • theoretical sampling • peer review
					Use of 'checklists' commonly recommended

**Process-oriented approach**	Internal, researcher-led; on-going	Critique of output-focused approach, with reliance on fixed techniques and constructs of quality derived from positivist paradigm	Principles or values of 'best practice', inherent to qualitative approach	Reflexivity Transparency Comprehensiveness Responsibility Ethical practice Systematic approach	Use of mechanisms which facilitate researcher's enactment of principles of quality, throughout research process, for example: • Use of field diary to reflect on position and assumptions • Audit trail to record methodological decisions made, for reflection at interpretation stage • Ensuring researchers' comprehension of and engagement with their role in assuring quality
					Recommending active methodological awareness over reliance on checklists of techniques

## Discussion

The literature reviewed showed a lack of consensus between qualitative research approaches about how to assure quality of research. This reflects past and on-going debates among qualitative researchers about how to define quality, and even the nature of qualitative research itself. The two main narratives that emerged from the reviewed literature reflected differing approaches to quality assurance and, underpinning these differing conceptualisations of quality in qualitative research.

Among the literature that directly discusses quality assurance in qualitative research, the most dominant narrative detected was that of an output-oriented approach. Within this narrative, quality is conceptualised in relation to theoretical constructs such as validity or rigour, derived from the positivist paradigm, and is demonstrated by the inclusion of certain recommended methodological techniques. By contrast, the second, process-oriented narrative presented conceptualisations of quality that were linked to principles or values considered inherent to the qualitative approach, to be understood and enacted throughout the research process. A third, minor narrative offered critique of current and recent discourses on assuring quality of qualitative research but did not appear to offer alternative ways by which to conceptualise or conduct quality assurance.

Strengths of the output-oriented approach for assuring quality of qualitative studies include the acceptability and credibility of this approach within the dominant positivist environment where decision-making is based on 'objective' criteria of quality [11]. Checklists equip those unfamiliar with qualitative research with the means to assess its quality [6]. In this way, qualitative research can become more widely accessible, accepted and integrated into decision-making. This has been demonstrated in the increasing presence of qualitative studies in leading medical research journals [11,12]. However, as argued by those contributing to the second narrative in this review, the following of check-lists does not equate with understanding of and commitment to the theoretical underpinnings of qualitative paradigms or what constitutes quality within the approach. The privileging of guidelines as a mechanism to demonstrate quality can mislead inexperienced qualitative researchers as to what constitutes good qualitative research. This runs the risk of reducing qualitative research to a limited set of methods, requiring little theoretical expertise [52] and diverting attention away from the analytic content of research unique to the qualitative approach [14]. Ultimately, one can argue that a solely output-oriented approach risks the values of qualitative research becoming skewed towards the demands of the positivist paradigm without retaining quality in the substance of the research process.

By contrast, strengths of the process-oriented approach include the ability of the researcher to address the quality of their research in relation to the core principles or values of qualitative research (see Table 2). For example, previous assumptions that incorporating participant-observation methods over an extended period of time in 'the field' constituted 'good' anthropology and an indicator of quality have been challenged on the basis that fieldwork as a method should not be conducted uncritically [53], without acknowledgement of other important steps, including exploring variability and contradiction [54], and being explicit about methodological choices made and the theoretical reasons behind them [55]. The core principles identified in this narrative also represent continuous, researcher-led activities, rather than externally-determined indicators such as validity, or end-points. Reflexivity, for example, is an active, iterative process [56], described as '*an attitude of attending systematically to the context of knowledge construction... at every step of the research process' *[p484, 23]. As such, this approach emphasises the need to consider quality throughout the whole course of research, and locates the responsibility for enacting good qualitative research practice firmly in the lap of the researcher(s).

The question remains, however, as to *how *researchers can demonstrate to others that core principles have guided their research process. The paucity of guidelines among those advocating a process-oriented approach suggests these are either not possible or not desirable to disseminate. Guidelines, by their largely fixed nature, could be considered incompatible with flexible, pluralistic, qualitative research. Awareness and understanding of the fundamental principles of qualitative research (such as those six identified in this review) could be considered sufficient to ensure that researchers conduct the whole research process to a high standard. Indeed, it could be argued that this type of approach has been promoted within qualitative research fields beyond the health sciences for several decades, since debates around how to do 'good' qualitative research emerged publically [41,43,51]. However, the premises of this approach are challenged by increasing scrutiny over the accuracy and ethics of the generation of information through scientific activity [57,58]. Previous critiques of a post-hoc evaluation approach to quality, in favour of procedural mechanisms to ensure good research [43], have not responded to the demand in some research contexts, particularly in global health, for externally demonstrable quality assurance procedures.

The authors propose, therefore, that some form of guidelines may be possible and desirable, although in a less structured format than those representing a more positivistic paradigm and based on researcher-led principles of good practice rather than externally-determined constructs of quality such as validity. However, first it is important to acknowledge some of the limitations of our search and interpretations.

### Limitations

The number of papers included in the review was relatively low. The search was limited to publications explicitly focused on 'quality assurance', and the inclusion criteria may have excluded relevant literature that uses different terminologies, particularly as this concept has not commonly been used within qualitative methods literature. As has been demonstrated in the narratives identified, approaches to quality assurance are linked closely to conceptualisations of quality, about which there is a much larger body of literature than was reviewed for this paper. The possibility of these publications being missed, along with other hard-to-find and grey literature, has implications for the robustness of the narratives identified.

This limitation is perhaps most evident in the lack of literature in this review identified from the field of anthropology. Debates around concepts such as validity and what constitutes 'knowledge' from research have long been of interest to anthropologists [55], but the absence of these in the publications which met the inclusion criteria raises questions about the search strategy used. Although the search strategy was revised iteratively during the search process to capture variations of quality assurance, anthropological references did not emerge. The choice was made not to pursue the search further for practical and time-related reasons, but also as we felt that limiting the review to quality assurance as originally described would be useful for understanding the literature that a researcher would likely encounter when exploring quality assurance of qualitative research. The lack of clear anthropological voice in this literature reflects the paucity of engagement with the theoretical basis of this discipline in the health sciences, unlike other social sciences such as sociology [52]. As such, anthropology's contributions to debates on qualitative research methods within health and medical research have been somewhat overlooked [59].

Hence, this review presents only a part of the discourse of assuring quality of qualitative research, but it does reflect the part that has dominated the fields of health and medical research. Although this review leaves some unanswered questions about defining and assuring quality across different qualitative disciplines, we believe it gives a valuable insight into the types of narratives a typical researcher would begin to engage with if coming from a global health research perspective.

### Recommendations

The narratives emerging from this literature review indicate the challenges related to approaching quality assurance from a perspective shaped by the positivist fields of evidence-based medicine, but also the lack of clear, structured guidance based on the intrinsic principles of qualitative research. We recommend that the strengths of both the output-oriented and process-oriented narratives be brought together to create guidance that reflects core principles of qualitative research but also responds to expectations of the global health field for explicitly assured quality in research. The fundamental principles characterising qualitative research, such as the six presented in Table 2, offer the basis of an approach to assuring quality that is reflexive of and appropriate to the specific values of qualitative research.

The next step in developing guidance should focus on identifying practical and specific advice to researchers as to how to engage with these principles and demonstrate enactment of the principles at each stage of the research process while being wary of promoting unthinking use of 'technical fixes' [6]. We recommend the development of a framework that helps researchers to identify their core principles, appropriate for their epistemological and methodological approach, and ways to demonstrate that these have been upheld throughout the research process. Current generic quality assurance activities, such as the use of standard operating procedures (SOPs) and monitoring visits could be attuned to the principles of the qualitative research being undertaken through an approach that demonstrates quality without constraining the research or compromising core principles. The development of such a framework should be undertaken in a collaborative way between researchers and field teams undertaking qualitative research in practice. We propose that this framework be flexible enough to accommodate different qualitative methodologies without dictating essential activities for promoting quality. Unlike previous guidance, we propose the framework should also respond to different demands from multi-disciplinary research teams and from external, positivist, audiences for evidence of quality assurance procedures, as may be faced, for example, in the field of global health research. This review has also highlighted the challenges of accessing a broad range of literature from across different social science disciplines (in particular anthropology) when conducting searches using standard approaches adopted in the health sciences. Further consideration should be taken as to how best to encourage wider search parameters, familiarisation with different sources of literature and greater acceptance of non-traditional disciplinary perspectives within health and medical literature reviews.

## Conclusions

Within the context of global health research, there is an increasing demand for the qualitative research field to move forwards in developing and establishing coherent mechanisms for quality assurance of qualitative research. The findings of this review have helped to clarify ways in which quality assurance has been conceptualised, and indicates a promising direction in which to take the next steps in this process. Yet, it also raises broader questions around how quality is conceptualised in relation to qualitative research, and how different qualitative disciplines and paradigms are represented in debates around the use of qualitative methods in health and medical research. We recommend the development of a flexible framework to help qualitative researchers to define, apply and demonstrate principles of quality in their research.

## Competing interests

The authors declare that they have no competing interests.

## Authors' contributions

JR helped with the design of the review, searched for and reviewed the literature and wrote the first draft of the manuscript. JK, NE, PM and EA contributed to the interpretation of the results and the writing the manuscript. CC conceived of the review and helped with its design, interpretation of results and writing the manuscript. All authors read and approved the final manuscript.
